# Valor Prognóstico do Colesterol não HDL na Pneumonia por COVID-19

**DOI:** 10.36660/abc.20220671

**Published:** 2023-06-01

**Authors:** Fatih Sivri, Mehtap Şencan, Şerife Barçın Öztürk, Ayşe Sema Maraşlı, Yahya Kemal İçen, Çağdaş Akgüllü

**Affiliations:** 1 Dortyol State Hospital Hatay Turquia Dortyol State Hospital, Hatay – Turquia; 2 Adnan Menderes University Aydın Turquia Adnan Menderes University, Aydın – Turquia; 3 Adana Health Practice and Research Center Adana Turquia Adana Health Practice and Research Center, Adana – Turquia

**Keywords:** Colesterol Não HDL/prognóstico, COVID-19, Pneumonia/fisiopatologia, Mortalidade

## Abstract

**Fundamento:**

Além da doença arterial coronariana, a lipoproteína de não alta densidade (não-HDL-C) fornece informações preditivas de curto e longo prazo para muitas doenças inflamatórias crônicas, como acidente vascular cerebral, hemodiálise, pós-transplante renal, hepatoesteatose não alcoólica e vírus da imunodeficiência humana.

**Objetivos:**

Este estudo examinou o valor preditivo do não-HDL-C medido antes do SARS-CoV-2 para mortalidade na infecção por COVID-19.

**Métodos:**

Este estudo incluiu retrospectivamente 1.435 pacientes diagnosticados com COVID-19 e tratados na enfermaria de doenças torácicas em um único centro entre janeiro de 2020 e junho de 2022. Todos os pacientes incluídos no estudo apresentavam características clínicas e radiológicas e sinais de pneumonia por COVID-19. O diagnóstico de COVID-19 de todos os pacientes foi confirmado por uma reação em cadeia da polimerase estudada a partir de um swab orofaríngeo. A significância estatística foi estabelecida em p < 0,05.

**Resultados:**

Os pacientes do estudo, incluindo 1.435 indivíduos, foram divididos em 712 pacientes no grupo de não sobreviventes e 723 no grupo de sobreviventes. Embora não tenha havido diferença entre os grupos em relação ao sexo, houve uma diferença de idade estatisticamente significativa. O grupo que não sobreviveu era mais velho. Idade, lactato desidrogenase (LDH), proteína C reativa (PCR), triglicerídeos, D-dímero e não-HDL-C foram fatores de risco independentes para mortalidade em análises de regressão. Na análise de correlação, idade, PCR e LDH foram positivamente correlacionados com não-HDL-C. Na análise ROC, a sensibilidade para não-HDL-C foi de 61,6% e a especificidade foi de 89,2%.

**Conclusão:**

Acreditamos que o nível de não HDL-C estudado antes da infecção por COVID-19 pode ser usado como um biomarcador prognóstico para a doença.

## Introdução

O COVID-19 tornou-se rapidamente uma pandemia global sem fim à vista. Apesar de numerosos estudos recentes para explicar os mecanismos celulares da doença, ainda existem questões não resolvidas. Pode levar à síndrome do desconforto respiratório agudo (SDRA), choque séptico, falência de múltiplos órgãos e até morte em pessoas com doença leve ou assintomática, especialmente em pacientes idosos com comorbidades. Além da idade, sexo, comorbidades e tratamentos médicos, vários biomarcadores demonstraram ter valor preditivo para prognóstico e mortalidade na doença de COVID-19.^1^

Embora os lipídios sejam os blocos básicos de construção das células e organelas, eles desempenham um papel na absorção, proliferação e transferência de materiais virais ou bacterianos para outras células.^2,3^ Ainda que os lipídios desempenhem um papel importante na penetração e disseminação da SARS- A infecção por CoV-2 em células, estudos e meta-análises geralmente investigaram as alterações lipídicas que ocorrem durante a infecção grave e o impacto dessas alterações no prognóstico da doença e na mortalidade.^4,5^

O colesterol de lipoproteína não de alta densidade (não-HDL-C) representa uma carga total de várias lipoproteínas aterogênicas: LDL-C, VLDL-C, IDL-C, Lp(a), remanescente de VLDL e remanescente de quilomícron. É considerado um melhor indicador do LDL-C, o alvo primário da aterosclerose. A principal vantagem do colesterol não HDL sobre o colesterol LDL é que ele contém remanescentes de VLDL e quilomícrons. Como o LDL-C, esses remanescentes de colesterol podem passar pela íntima vascular e causar aterosclerose.^6^ Meta-análises demonstraram que a carga aterosclerótica do C não-HDL é melhor do que a do colesterol LDL, especialmente na hipertrigliceridemia leve a moderada.^7^ Além disso, muitos estudos de prevenção primária e secundária mostraram que o não-HDL-C reflete melhor a aterosclerose do que o LDL-C, independentemente de fatores de risco, como idade, sexo e diabetes.^8^

Além de doença arterial coronariana, acidente vascular cerebral, hemodiálise, transplante renal, hepatoesteatose não alcoólica e síndrome da apneia obstrutiva do sono (SAOS), fornece informações preditivas de curto e longo prazo para muitas doenças inflamatórias crônicas.^9-13^ Além disso, o não-HDL-C é conhecido por prever a gravidade, comorbidade e mortalidade de várias infecções virais. Um colesterol não HDL alto é um fator de risco independente para a rápida deterioração da função renal e aterosclerose subclínica em indivíduos infectados pelo vírus da imunodeficiência humana (HIV).^14^ Um estudo multicêntrico, prospectivo e observacional de Levy et al. encontraram uma associação entre não-HDL-C alto, baixa contagem de CD4 e alta carga viral em idosos infectados pelo HIV.^15^

Este estudo examinou o valor preditivo do não-HDL-C medido antes do SARS-CoV-2 para mortalidade na infecção por COVID-19.

## Métodos

### Desenho do estudo e pacientes

Este estudo avaliou 2.052 pacientes tratados com infecção por COVID-19 em condições hospitalares entre janeiro de 2020 e junho de 2022. Insuficiência renal, neoplasias, doença do tecido colágeno e pacientes recebendo estatina e/ou terapia hipolipemiante foram excluídos do estudo. Os níveis lipídicos padrão (colesterol total (TC), lipoproteína de alta densidade (HDL-C), LDL-C, triglicerídeos (TG), não-HDL-C) dos 1.435 dos 1.750 pacientes restantes após os critérios de exclusão, foram obtidos nos cinco anos (tempo médio: 3,1 anos) anteriores à infecção por COVID-19. O estudo foi um estudo observacional retrospectivo para o qual foi obtida a aprovação do comitê de ética local. A seleção do grupo de estudo está resumida na Figura Central.

**Figura Central:**
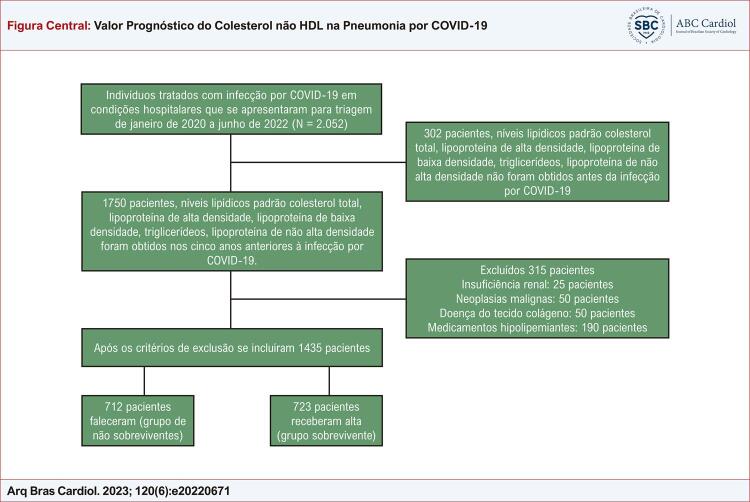
: Valor Prognóstico do Colesterol não HDL na Pneumonia por COVID-19

Diagrama mostra a seleção dos grupos de estudo.

Todos os pacientes incluídos no estudo apresentavam características clínicas e radiológicas e sinais de pneumonia por COVID-19. O diagnóstico de COVID-19 foi confirmado por reação em cadeia da polimerase (PCR) com swab orofaríngeo. Todos os pacientes foram minuciosamente avaliados para hipertensão (HT), diabetes mellitus (DM), tabagismo, infarto do miocárdio prévio, insuficiência renal crônica (IRC), insuficiência cardíaca congestiva (ICC), doença pulmonar obstrutiva crônica (DPOC), evento cerebrovascular (ECV ).

Primeiro segundo da expiração forçada (VEF1) / Capacidade vital forçada (FEVC) < 70% ou VEF1 < 70% após broncodilatador inalatório foram aceitos como critérios diagnósticos para DPOC. Fração de ejeção (FE) < 35% foi considerada ICC por causas isquêmicas ou não isquêmicas. Uma taxa de filtração glomerular inferior a 60 em três meses foi tomada como CRF. O diagnóstico de HT foi feito se os pacientes estivessem recebendo tratamento anti-hipertensivo ou tivessem pressão arterial sistólica superior a 140 mmHg e pressão arterial diastólica superior a 90 mmHg em pelo menos três medições. Pacientes em uso de medicação antidiabética ou com pelo menos duas medições de glicemia de jejum superiores a 126 mg/dl foram classificados como DM.

### Exame ecocardıográfıco

O exame ecocardiográfico de todos os pacientes incluídos no estudo foi realizado com o sistema de ultrassom cardíaco iE33 (Phillips Healthcare, Best, Holanda) e um sistema de sonda de 2,5-5 MHz. A FE foi medida com o método de Simpson modificado.

### Análıse laboratorıal

Os valores basais hematológicos e bioquímicos de todos os pacientes incluídos no estudo foram recuperados do sistema eletrônico e registrados. Além disso, os biomarcadores séricos (D-dímero, lactato desidrogenase (LDH)) associados ao prognóstico da infecção por COVID-19 foram determinados na hospitalização inicial. Antes da infecção por COVID-19, os níveis lipídicos padrão dos pacientes, LDL-C, HDL-C, TC, não-HDL-C e TG, foram escaneados e registrados. O não-HDL-C foi medido subtraindo-se o valor de HDL-C do TC. O valor do LDL-C foi calculado de acordo com a fórmula de Friedewald.

### Análise estatística

Os pacotes estatísticos IBM SPSS Statistics for Windows (versão 25.0) (NY, EUA) e Amos (versão 24.0) (WA, EUA) foram usados para analisar os dados. O teste de Kolmogorov-Smirnov avaliou se os dados tinham uma distribuição normal. Variáveis contínuas são apresentadas como média (desvio padrão) se a variável for paramétrica ou mediana (intervalo interquartil: Q1 a Q3) se a variável for distribuída como valores não paramétricos. As variáveis foram comparadas com valores independentes do teste t ou do teste de Mann-Whitney, dependendo do tipo de distribuição dos dados. Variáveis categóricas são apresentadas como números e porcentagens. Os testes qui-quadrado e exato de Fisher foram realizados para comparar variáveis categóricas. A relação entre as duas variáveis contínuas foi avaliada por meio do coeficiente de correlação de Pearson e, quando as condições para o teste paramétrico não foram atendidas, foi calculado o coeficiente de correlação de Spearman. Um nível de p<0,05 foi considerado estatisticamente significativo. As variáveis para as quais o valor de p não ajustado foi <0,05 no modelo de regressão logística foram identificadas como potenciais marcadores de risco e incluídas no modelo multivariado completo. Foram utilizadas análises de regressão logística multivariada de eliminação retrógrada usando um teste de razão de verossimilhança para eliminar variáveis. A curva de características operacionais do receptor foi usada para determinar a sensibilidade e especificidade do não-HDL-C e o valor de corte ideal para prever a mortalidade por COVID-19.

## Resultados

Os dados sociodemográficos de 1.435 pacientes, incluindo aqueles que concordaram em participar do estudo, foram os seguintes: 712 pacientes foram a óbito (grupo não sobreviventes) e 723 pacientes receberam alta (grupo sobreviventes). Embora não tenha havido diferença entre os grupos sobreviventes e não sobreviventes em relação ao sexo, houve uma diferença de idade estatisticamente significativa. O grupo que não sobreviveu era mais velho. (Tabela 1-2)

Quando os grupos foram avaliados quanto aos fatores de risco e doenças adicionais, não foi encontrada diferença no grupo não sobrevivente em relação ao grupo sobrevivente. (Tabela 1)

**Tabela 1:** – Comparação das características clínicas dos pacientes não sobreviventes e sobreviventes

		Grupo				p

		Não sobreviventes (n=712)		Sobreviventes (n=723)		

		n	%	n	%	
Gênero	Masc.	437	61.4	432	59,8	0,529
	Fem.	275	38,6	291	40.2	
HT	Não	204	28,7	273	37,8	0,558
	Sim	508	71.3	450	62,2	
DM	Não	430	60,4	446	61,7	0,615
	Sim	282	39,6	277	38.3	
ECV	Não	620	87.1	654	90,5	0,122
	Sim	92	12.9	69	9.5	
ICC	Não	466	65,4	503	69,6	0,255
	Sim	246	34,6	220	30,4	
DPOC	Não	398	55,9	473	64,5	0,435
	Sim	314	44.1	250	35,5	

Os valores são mostrados em número e percentil. HT: hipertensão; DM: diabetes mellitus; ECV: evento cerebrovascular; ICC: insuficiência cardíaca crônica; DPOC: doença pulmonar obstrutiva crônica.

Ao comparar os grupos quanto aos valores laboratoriais, as plaquetas e o HDL-C foram maiores no grupo sobrevivente do que no grupo não sobrevivente. LDH, proteína C reativa (PCR), glóbulo branco (WBC), dímero D, TG e não-HDL-C foram estatisticamente maiores no grupo não sobrevivente do que no grupo sobrevivente. Não houve diferença estatisticamente significativa entre os grupos quanto aos valores de sódio. No entanto, hiponatremia foi observada em ambos os grupos (Tabela 2)

**Tabela 2:** – Comparação dos parâmetros laboratoriais dos pacientes nos grupos não sobreviventes e sobreviventes

	Grupo		p

	Não sobreviventes (n=712)	Sobreviventes (n=723)	
Idade	64,25±35,2	50,14±47,4	0,000
Creatinina	1,0 (0,2-9,0)	1,0 (0,3-1,6)	0,271
LDH	527,0 (47,0-6500,0)	263,0 (42,0-2500,0)	0,000
Sódio	132,0 (116,0-143,0)	131,0 (125,0-141,0)	0,269
Potássio	4,0 (2,9-7,1)	4,0 (2,1-5,1)	0,276
PCR	126,0 (2,3-400,0)	51,0 (0,2-423,0)	0,000
Glóbulos brancos	11,8 (1,2-102,0)	9,5 (2,1-96,0)	0,001
HB	11,8 (5,8-17,8)	12,0 (3,5-18,6)	0,075
PLT	208,0 (7,9-600,0)	243,0 (18,0-890,0)	0,000
D-dímero	4,9 (0,1-136,0)	1,5 (0,1-29,1)	0,000
Troponina	83,0 (0,0-4000,0)	50,0 (0,1-3750,0)	0,095
CT	200,0 (51,0-389,0)	200,0 (1,0-485,0)	0,097
LDL-C	120 (9,0-231,0)	112,0 (18,0-293,0)	0,809
HDL-C	34,0 (3,0-120,0)	48,0 (10,0-144,0)	0,000
TG	156,0 (42,0-1222,0)	146,0 (90,0-2000,0)	0,007
Não HDL-C	155,0 (15,0-361,0)	146,0 (98,0-451,9)	0,000

Os valores são apresentados como média ± desvio padrão, mediana e intervalo interquartílico. LDH: lactato desidrogenase; PCR: proteína C reativa; HB: hemoglobina PLT: plaquetas; CT: colesterol total; LDL-C: colesterol de baixa densidade; HDL-C: colesterol de alta densidade; TG: triacilglicerol; não HDL-C: colesterol não de alta densidade.

**Figura 1:**
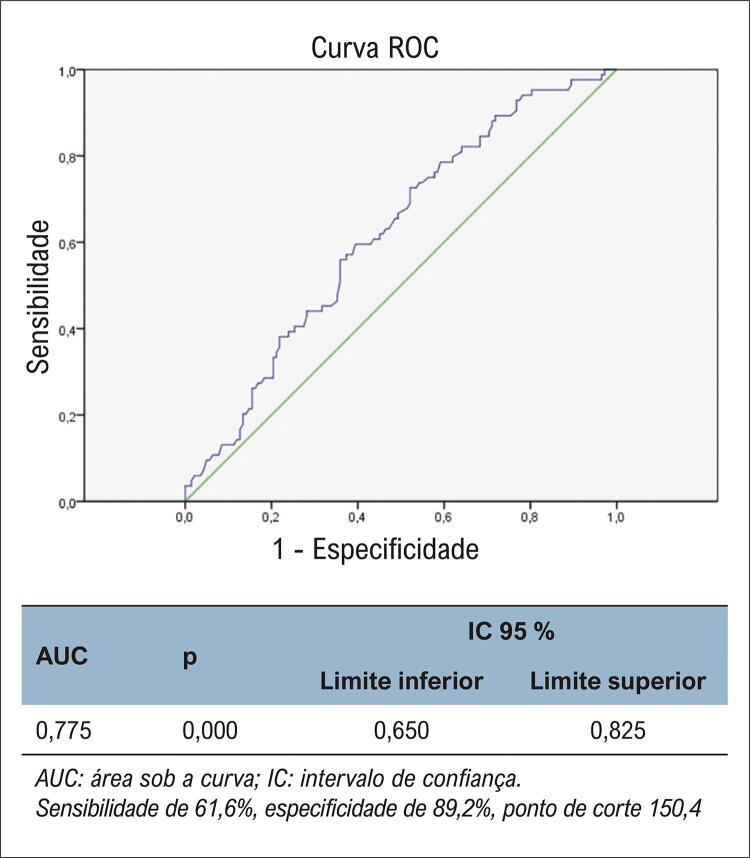
– Análise da curva ROC do colesterol não HDL.

AUC: área sob a curva; IC: intervalo de confiança. Sensibilidade de 61,6%, especificidade de 89,2%, ponto de corte 150,4

Idade, LDH, PCR, D-dímero, TG e não-HDL-C foram considerados fatores de risco independentes para mortalidade em análises de regressão univariadas e multivariadas. (Tabela 3) Na análise de correlação de Pearson e Spearman, idade, PCR e LDH foram positivamente correlacionados com não-HDL-C. (Tabela 4) Na análise ROC, a sensibilidade para não-HDL-C foi de 61,6% e a especificidade foi 89,2%. (Figura 1)

**Tabela 3:** – Impacto de diferentes variáveis no grupo de não sobreviventes em análises de regressão logística univariada e multivariada

	Univariado				Multivariada			
	OR	IC 95%		p	OR	IC 95%		p
Idade	1,655	1,256	2,122	0,000	1,588	1,352	1,755	0,000
LDH	1,004	1,004	1,005	0,000	1,002	1,002	1,003	0,000
PCR	1,010	1,009	1,012	0,000	1,004	1,002	1,006	0,000
Glóbulos brancos	1,057	1,039	1,076	0,000	0,965	0,928	1,003	0,072
TG	1,001	1,000	1,002	0,032	1,005	1,002	1,010	0,03
D-dímero	1,259	1,214	1,306	0,000	1,131	1,088	1,175	0,000
Não HDL-C	1,004	1,002	1,006	0,000	1,005	1,002	1,008	0,001

IC: intervalo de confiança; OR: razão de chances; p: valor p; LDH: lactatodesidrogenase; PCR: Proteína C reativa; TG: triglicerídeo; não HDL-C: colesterol não de alta densidade.

**Tabela 4:** – Correlação entre não-HDL-C e biomarcadores séricos

N:712		Não HDL-C
Idade	r	0,333
	p	0,001
LDH	r	0,222
	p	0,002
PCR	r	0,235
	p	0,025

LDH: lactato desidrogenase; PCR: Proteína C reativa.

## Discussão

Nosso estudo é o primeiro a investigar a associação entre a infecção por SARS-COV-2 e o nível não-HDL-C estudado antes da doença COVID-19. A primeira descoberta deste estudo é que o nível de não-HDL-C estudado antes da doença COVID-19 é um fator de risco independente para mortalidade. O segundo achado importante é que o nível de não-HDL-C se correlaciona positivamente com a idade, PCR e LDH.

Semelhante a estudos anteriores, nosso estudo identificou idade, TG, LDH, PCR e D-dímero como fatores de risco independentes para infecção por COVID-19.^16^ Aumento de comorbidade, fragilidade e distúrbios do sistema imunológico que aumentam com a idade são considerados fatores importantes para o prognóstico da infecção por COVID-19.^17^ No estudo conduzido por Onder G et al.,^18^ na Itália, a taxa de mortalidade em pacientes hospitalizados com infecção por COVID-19 foi de 0,4% nos menores de 40 anos, 0,4% nos 50 anos, 3,5% nos 60 anos, 12,8% nos 70 anos e 20,2% naqueles com 80 anos ou mais.18 Embora nenhuma diferença de gênero tenha sido observada entre os grupos em nosso estudo, foi observado em estudos epidemiológicos que a gravidade e a mortalidade da infecção por COVID-19 são maiores, especialmente em homens mais velhos.^19^ No estudo epidemiológico de Zou et al.,^20^ a média de idade dos falecidos foi de 56 anos, sendo a maioria do sexo masculino (70%). aumento de mediadores oxidativos e inflamatórios (TNF-alfa, IL-1, IL-6) e disfunção do sistema imunológico. Além disso, estudos epidemiológicos constataram que o grupo de pacientes do sexo masculino tem menor conhecimento da doença e adesão ao tratamento. Quando consideradas as doenças concomitantes, verifica-se que o tabagismo é mais prevalente no sexo masculino, e as doenças cardiovasculares e respiratórias decorrentes causam alta mortalidade.^21^

A PCR resultante da liberação de IL-6 é um fator de risco independente tanto para o prognóstico quanto para a mortalidade da doença.^22^ Smilowitz et al.,^23^ em seu estudo com 2.872 pacientes, constataram que o risco de tromboembolismo venoso aumentou 2,33 vezes, o risco de insuficiência renal aguda 2,11 vezes e risco de mortalidade 2,59 vezes maior em pacientes com PCR elevada. A LDH é uma enzima intracelular encontrada em quase todos os sistemas de órgãos.^23^ Níveis elevados de LDH foram observados em infecções por COVID-19, aumentando a gravidade da doença em 6 vezes e a mortalidade em 16 vezes.^24^ O dímero D é um importante biomarcador da coagulação sanguínea e fibrinólise que aumenta significativamente na coagulação intravascular disseminada (CIVD). Estudos demonstraram uma estreita associação entre D-dímeros e a gravidade e mortalidade da doença. No estudo de Zhang L. et al. em pacientes tratados no hospital, a mortalidade foi maior em pacientes com dímero D > 2µg/mL com sensibilidade de 92% e especificidade de 83,3%.^25^ Embora não tenha havido diferença estatística nos valores de sódio entre os grupos em nosso estudo, leve hiponatremia foi detectada em ambos os grupos. Em uma metanálise de 23 estudos sobre infecções por COVID-19, a hiponatremia foi observada em 25,8% de 38.753 pacientes e foi mais comum em pacientes tratados em hospitais e unidades de terapia intensiva.^26^Habas et al.,^27^ relataram que a hiponatremia é muito comum em pacientes com infiltração pulmonar radiológica e que a gravidade da doença é diretamente proporcional à profundidade da hiponatremia.^27^ Acreditamos que a razão para a hiponatremia observada em ambos os grupos em nosso estudo seja a população de pacientes. Basicamente, assumiu-se que todos os pacientes incluídos no estudo apresentavam infiltração pulmonar radiológica e foram tratados em condições hospitalares.

Embora o não-HDL-C mostre uma carga aterogênica em estudos recentes, demonstrou ter valor preditivo e prognóstico para muitas doenças crônicas inflamatórias, como a doença arterial coronariana, a hepatoesteatose não alcoólica, SAOS, HIV e hepatite B (HBV).^9-13^ O processo inflamatório começa com a formação de macrófagos carregados de lipídios pró-inflamatórios (foam) passando pela camada endotelial vascular de não-HDL-C. Isso leva à peroxidação lipídica e à formação de radicais livres de oxigênio. Esses produtos formados ativam fatores de transcrição semelhantes ao fator nuclear (NF) κB e causam a liberação de citocinas inflamatórias (TNF-alfa e IL-1B). Esses mecanismos causam o início e a progressão da inflamação vascular. Numerosos estudos relataram que a elevação lipídica aterosclerótica desencadeia inflamação sistêmica e local.^28^ Em um estudo animal realizado por Busnelli et al.,^29^ foi demonstrado que uma dieta hipercolesterolêmica desencadeia inflamação sistêmica vascular e crônica. Como resultado do estudo, além de aumento de leucócitos, monócitos e linfócitos plasmáticos em vários sistemas, como fígado e tecido adiposo branco, aumento de macrófagos e linfócitos de células T, bem como aumento de citocinas inflamatórias (TNF-alfa, IL-1B , IL-6), foram observados.^29^ Wang et al.,^30^ relataram que o não-HDL-C é um marcador precoce de disfunção endotelial vascular em pacientes com DM tipo 2 e se correlaciona com a PCR.^30^ Prado et al.,^31^ mostraram em seu estudo que o não-HDL-C é um melhor indicador de progressão da doença e controle glicêmico do que a PCR em adolescentes e crianças com DM tipo 1.^31^ No estudo animal conduzido por Poledne et al.,^32^ foi encontrada uma correlação positiva entre o desenvolvimento de macrófagos pró-inflamatórios (CD14-16-36) no tecido adiposo visceral, que forma a base para o desenvolvimento da síndrome metabólica e aterosclerose, e não-HDL-C.^32^ Cippollene et al.,^33^ descobriram que o não-HDL-C era maior em pacientes que desenvolveram reestenose após angioplastia coronária transluminal percutânea, e houve correlação positiva entre reestenose e IL-1B e PCR.^33^ No estudo de Karasek et al.,^34^ foi observada correlação positiva entre não HDL-C e os marcadores inflamatórios PCR, peptídeo C e PAI ( inibidor da ativação do plasminogênio).^34^ De acordo com os resultados de um estudo de acompanhamento de 4 anos em pacientes crônicos com HBV, Joo et al.,^35^ relataram que não-HDL-C foi 0,69 vezes maior em pacientes HBsAg-positivos do que em pacientes negativos.^35^

Embora nenhum estudo direto examine a relação entre sepse e não-HDL-C, os níveis de PCSK-9, que formam a base do metabolismo de não-HDL-C, são considerados marcadores importantes de sepse. A PCSK-9 é uma serina protease sintetizada principalmente no fígado. Sua principal função é degradar os receptores de LDL na superfície do fígado. Além disso, destrói o receptor da apolipoproteína E, receptor Toll-like, receptor VLDL e proteína relacionada ao LDL-1 (LDLR1), o que pode levar a um aumento anormal na concentração plasmática de lipoproteínas e níveis de citocinas e trombose.^36^ Muitos estudos têm encontrado um aumento significativo nos níveis de PCSK-9 em pacientes com sepse e/ou choque séptico.^37^ Walley et al.,^38^ investigaram o efeito da perda de função (LOF) e ganho de função de PCSK-9 na mortalidade em humanos e camundongos em choque séptico. Foi determinado que LOF no grupo humano reduziu a mortalidade no dia 28 (61% versus 71%). Além disso, quando avaliado após 72 horas no grupo de camundongos, verificou-se que a formação de citocinas inflamatórias e endotoxinas foi menor no grupo com LOF.^38^ Boyd et al.,^39^ mostraram níveis elevados de PCSK-9 durante a sepse e formação aumentada de endotoxina bacteriana fortemente correlacionada com falência de múltiplos órgãos.^39^ Embora não haja informações claras entre pneumonia por COVID-19 e PCSK-9, previu-se que a inibição de PCSK-9 pode reduzir a infecciosidade viral, como demonstrado por várias hipóteses.^40^

No estudo de Mostaza et al.,^41^ que examinou o efeito prognóstico dos níveis lipídicos antes da infecção por COVID-19, foi encontrada uma associação inversa entre um alto nível de HDL-C e mortalidade em indivíduos mais velhos antes da doença.^41^ Este é um status semelhante ao nosso estudo. Em outro estudo de Masana et al., foram examinados os níveis de lipídios séricos de 1.305 pacientes. Ao final do estudo, embora a infecção por COVID-19 fosse mais grave em pacientes com TG alto e HDL-C baixo, nenhum efeito prognóstico do C-não-HDL foi observado.^42^ Essa situação está em contradição com nosso estudo. Em nosso estudo, níveis elevados de não-HDL-C, juntamente com níveis elevados de TG e baixo nível de HDL-C, foram considerados um fator de risco independente para mortalidade. Suspeitamos que isso se deva às diferenças na população de pacientes e no método de estudo entre os estudos. Em seu estudo, Masana et al.,^42^ indicaram que IRC, câncer e DM, considerados fatores de risco independentes para mortalidade na infecção por COVID-19, foram altos no grupo de infecção grave. Além disso, níveis não-HDL-C e uso de estatina, que têm valor preditivo para mortalidade na infecção por COVID-19, não foram investigados entre os grupos. No entanto, nosso estudo não incluiu pacientes em uso de estatinas ou pacientes com câncer e IRC, que são fatores de risco independentes para mortalidade na infecção por COVID-19.

### Limitações

As principais limitações do estudo são as seguintes: é baseado em um único centro e desenho de estudo retrospectivo, o mecanismo exato dessa relação não pôde ser elucidado com precisão e pôde ter ocorrido viés na seleção de grupos de controle. Além disso, as alterações lipídicas durante a primeira internação dos pacientes e após o tratamento não puderam ser examinadas. Os tratamentos administrados para a infecção por COVID-19 não foram estudados. Os níveis de apolipoproteína B, que indicam carga aterogênica total em vez de não-HDL-C, não foram medidos. Outra limitação do estudo é que o acompanhamento de curto e longo prazos não foi realizado.

## Conclusão

Nosso estudo mostrou pela primeira vez que um nível mais alto de não-HDL-C antes da infecção por COVID-19 é um fator de risco independente para mortalidade. Acreditamos que o nível de não HDL-C estudado antes da infecção por COVID-19 pode ser usado como um biomarcador prognóstico para a doença. Além disso, acreditamos que pode nos ajudar a entender a fisiopatologia e desenvolver novas estratégias de tratamento. Mais estudos prospectivos com grandes amostras são necessários para entender melhor a patogênese do COVID-19 e o valor diagnóstico e terapêutico do não-HDL-C em pacientes com COVID-19.
