# Supplementary material for: Valor Prognóstico do Colesterol não HDL na Pneumonia por COVID-19
Source: Arq Bras Cardiol. 2023 Jun 1;120(6):e20220671. [Article in Portuguese] doi: 10.36660/abc.20220671 (PMC10263406; doi:10.36660/abc.20220671)
Supplement: Translation

# Translation: Prognostic Value of Non-HDL Cholesterol in COVID-19 Pneumonia

**Authors:** Fatih Sivri, Mehtap Şencan, Şerife Barçın Öztürk, Ayşe Sema Maraşlı, Yahya Kemal İçen, Çağdaş Akgüllü

**Affiliations:** 1 Turkey Dortyol State Hospital, Hatay – Turkey; 2 Turkey Adnan Menderes University, Aydın – Turkey; 3 Turkey Adana Health Practice and Research Center, Adana – Turkey

**Keywords:** HDL-C/prognosis, COVID-19, Pneumonia/physiopathology, Mortality

## Abstract

**Background:**

In addition to coronary artery disease, non-high-density lipoprotein(non-HDL-C) provides short and long-term predictive information for many chronic inflammatory diseases such as stroke, hemodialysis, post-renal transplant, non-alcoholic hepatosteatosis, and human immunodeficiency virus.

**Objectives:**

This study examined the predictive value of non-HDL-C measured before SARS-CoV-2 for mortality in COVID-19 infection.

**Methods:**

This study retrospectively included 1435 patients diagnosed with COVID-19 and treated in the thoracic diseases ward in a single center between January 2020 and June 2022. All patients included in the study had clinical and radiological features and signs of COVID-19 pneumonia. The COVID-19 diagnosis of all patients was confirmed by a polymerase chain reaction studied from an oropharyngeal swab. Statistical significance was set at p < 0.05.

**Results:**

The study patients, including 1435 subjects, were divided into 712 patients in the non-surviving group and 723 in the surviving group. While there was no difference between the groups regarding gender, there was a statistically significant age difference. The non-surviving group was older. Age, lactate dehydrogenase(LDH), C reactive protein(CRP), triglycerides, D-dimer, and non-HDL-C were independent risk factors for mortality in regression analyses. In correlation analysis, age, CRP, and LDH were positively correlated with non-HDL-C. In the ROC analysis, sensitivity for non-HDL-C was 61.6%, and specificity was 89.2%.

**Conclusion:**

We believe that the non-HDL-C level studied before COVID-19 infection can be used as a prognostic biomarker for the disease.

## Introduction

COVID-19 has rapidly become a global pandemic with no end in sight. Despite numerous recent studies to explain the cellular mechanisms of the disease, there are still unresolved questions. It can lead to acute respiratory distress syndrome (ARDS), septic shock, multiorgan failure, and even death in persons with mild or asymptomatic disease, especially in elderly patients with comorbid diseases. In addition to age, gender, comorbid disease, and medical treatments, several biomarkers have been shown in studies to have predictive value for prognosis and mortality in COVID-19 disease.^1^

Although lipids are the basic building blocks of cells and organelles, they play a role in the uptake, proliferation, and transfer of viral or bacterial materials to other cells.^2,3^ Although lipids play an important role in the penetration and spread of SARS-CoV-2 infection into cells, studies, and meta-analyses have generally investigated the lipid changes that occur during severe infection and the impact of these changes on disease prognosis and mortality.^4,5^

Non-high-density lipoprotein cholesterol (non-HDL-C) represents a total burden of several atherogenic lipoproteins: LDL-C, VLDL-C, IDL-C, Lp(a), VLDL remnant, and chylomicron remnant. It is considered a better indicator of LDL-C, the primary target of atherosclerosis. The main advantage of non-HDL cholesterol over LDL cholesterol is that it contains VLDL and chylomicron remnants. Like LDL-C, these remnants of cholesterols can pass through the vascular intima and cause atherosclerosis.^6^ Meta-analyses have shown that the atherosclerotic burden of non-HDL-C is better than that of LDL cholesterol, especially in mild to moderate hypertriglyceridemia.^7^ Also, many primary and secondary prevention studies have shown that non-HDL-C reflects atherosclerosis better than LDL-C, independent of risk factors such as age, gender, and diabetes.^8^

In addition to coronary artery disease, stroke, hemodialysis, renal transplantation, non-alcoholic hepatosteatosis, and obstructive sleep apnea syndrome (OSAS), it provides short- and long-term predictive information for many chronic inflammatory diseases.^9-13^Moreover, non-HDL-C is known to predict the severity, comorbidity, and mortality of several viral infections. A high non-HDL-C is an independent risk factor for the rapid deterioration of renal function and subclinical atherosclerosis in human immunodeficiency virus(HIV) infected individuals.^14^ A multicenter, prospective, observational study by Levy et al. found an association between a high non-HDL-C, low CD4 count, and high viral load in HIV-infected elderly individuals.^15^

This study examined the predictive value of non-HDL-C measured before SARS-CoV-2 for mortality in COVID-19 infection.

## Methods

### Study design and patients

This study evaluated 2052 patients treated with COVID-19 infection under hospital conditions between January 2020 and June 2022. Renal insufficiency, malignancies, collagen tissue disease, and patients receiving statin and/or lipid-lowering therapy were excluded from the study. Standard lipid levels (total cholesterol (TC), high-density lipoprotein (HDL-C), LDL-C, triglycerides (TG), non-HDL-C) of the 1,435 of the 1,750 patients remaining after the exclusion criteria were met were obtained in the five years (average time: 3.1 years) before COVID-19 infection. The study was a retrospective observational study for which approval was gathered from the local ethics committee. The selection of the study group is summarized in the central illustration.

**Central Illustration:** : Prognostic Value of Non-HDL Cholesterol in COVID-19 Pneumonia

Diagram shows the selection of the study groups.

All patients included in the study had clinical and radiological features and signs of COVID-19 pneumonia. The diagnosis of COVID-19 was confirmed by polymerase chain reaction (PCR) with an oropharyngeal swab. All patients were thoroughly evaluated for hypertension (HT), diabetes mellitus (DM), tobacco use, previous myocardial infarction, chronic renal failure (CRF), congestive heart failure (CHF), chronic obstructive pulmonary disease (COPD), cerebrovascular event (CVE).

First second of forced expiration (FEV1) / Forced vital capacity (FEVC) < 70% or FEV1 < 70% after inhaled bronchodilator were accepted as diagnostic criteria for COPD. Ejection fraction (EF) < 35% was regarded as CHF due to ischemic or non-ischemic causes. A glomerular filtration rate of less than 60 over three months was taken as CRF. The diagnosis of HT was made if patients were receiving antihypertensive treatment or had a systolic blood pressure of more than 140 mmHg and diastolic blood pressure of more than 90 mmHg on at least three measurements. Patients taking antidiabetic medication or having at least two fasting blood glucose measurements greater than 126 mg/dl were classified as DM.

### Echocardiographic examination

Echocardiographic examination of all patients included in the study was performed with the iE33 cardiac ultrasound system (Phillips Healthcare, Best, The Netherlands) and a 2.5-5-MHz probe system. The EF was measured with the modified Simpson method.

### Laboratory analysis

The baseline hematological and biochemical values of all patients enrolled in the study were retrieved from the electronic system and recorded. In addition, serum biomarkers (D-dimer, lactate dehydrogenase (LDH)) associated with the prognosis of COVID-19 infection were determined at initial hospitalization. Before COVID-19 infection, patients’ standard lipid levels, LDL-C, HDL-C, TC, non-HDL-C, and TG, were scanned and recorded. Non-HDL-C was measured by subtracting the HDL-C value from TC. The LDL-C value was calculated according to the Friedewald formula.

### Statistical analysis

IBM SPSS Statistics for Windows (version 25.0) (NY, USA) and Amos (version 24.0) (WA, USA) statistical packages were used to analyze the data. The Kolmogorov–Smirnov test assessed whether the data had a normal distribution. Continuous variables are presented as mean (standard deviation) if the variable is parametric or median (interquartile range: Q1 to Q3) if the variable is distributed as non-parametric values. Variables were compared with independent t-test or Mann–Whitney test values depending on the type of data distribution. Categorical variables are presented as numbers and percentages. The chi-square and Fisher’s exact tests were performed to compare categorical variables. The relationship between the two continuous variables was assessed using the Pearson correlation coefficient, and when the conditions for the parametric test were not met, the Spearman correlation coefficient was calculated. A p<0.05 level was considered statistically significant. The variables for which the unadjusted *p-*value was <0.05 in the logistic regression model were identified as potential risk markers and included in the full multivariate model. Backward elimination multivariate logistic regression analyses using a likelihood ratio test to eliminate variables were utilized. The receiver operating characteristics curve was used to determine the sensitivity and specificity of non-HDL-C and the optimal cutoff value for predicting COVID-19 mortality.

## Results

The sociodemographic data of 1435 patients, including those who agreed to participate in the study, were as follows: 712 patients were deceased (non-surviving group), and 723 patients were discharged (surviving group). While there was no difference between the surviving and non-surviving groups regarding gender, there was a statistically significant age difference. The non-surviving group was older. (Table 1-2)

When the groups were evaluated regarding risk factors and additional diseases, no difference was found in the non-surviving group than in the surviving group. (Table 1)

**Table 1:** – Comparison of patient clinical characteristics in the non-surviving and surviving groups

		Group				p

		Non-surviving (n=712)		Surviving (n=723)		

		n	%	n	%	
Gender	Male	437	61.4	432	59.8	0.529
	Female	275	38.6	291	40.2	
HT	No	204	28.7	273	37,8	0.558
	Yes	508	71.3	450	62,2	
DM	No	430	60.4	446	61.7	0.615
	Yes	282	39.6	277	38.3	
CVE	No	620	87.1	654	90.5	0.122
	Yes	92	12.9	69	9.5	
CHF	No	466	65.4	503	69,6	0,255
	Yes	246	34.6	220	30,4	
COPD	No	398	55.9	473	64,5	0.435
	Yes	314	44.1	250	35,5	

Values are shown in number and percentile. HT: hypertension; DM: diabetes mellitus; CVE: cerebrovascular event; CHF: chronic heart failure; COPD: chronic obstructive pulmonary disease.

When comparing the groups regarding laboratory values, platelets and HDL-C were higher in the surviving group than in the non-surviving group. LDH, C reactive protein (CRP), white blood cell (WBC), D-dimer, TG, and non-HDL-C were statistically higher in the non-surviving group than in the surviving group. No statistically significant difference was found between the groups regarding sodium values. However, hyponatremia was observed in both groups (Table 2) Age, LDH, CRP, D-dimer, TG, and Non-HDL-C were found to be independent risk factors for mortality in univariate and multivariate regression analyses. (Table 3) In Pearson and Spearman correlation analysis, age, CRP, and LDH were positively correlated with non-HDL-C.(Table 4) In the ROC analysis, sensitivity for non-HDL-C was 61.6%, and specificity was 89.2%. (Figure 1).

**Table 2:** – Comparison of patients’ laboratory parameters in the non-surviving and surviving groups

	Group		p

	Non-surviving(n=712)	Surviving (n=723)	
Age	64.25±35.2	50.14±47.4	0.000
Creatinin	1.0 (0.2-9.0)	1.0 (0.3-1.6)	0.271
LDH	527.0 (47.0-6500.0)	263.0 (42.0-2500.0)	0.000
Sodium	132.0 (116.0-143.0)	131.0 (125.0-141.0)	0.269
Potassium	4.0 (2.9-7.1)	4.0 (2.1-5.1)	0.276
CRP	126.0 (2.3-400.0)	51.0 (0.2-423.0)	0.000
WBC	11.8 (1.2-102.0)	9.5 (2.1-96.0)	0.001
HB	11.8 (5.8-17.8)	12.0 (3.5-18.6)	0.075
PLT	208.0 (7.9-600.0)	243.0 (18.0-890.0)	0.000
D-dimer	4.9 (0.1-136.0)	1.5 (0.1-29.1)	0.000
Troponin	83.0 (0.0-4000.0)	50.0 (0.1-3750.0)	0.095
TC	200.0 (51.0-389.0)	200.0 (1.0-485.0)	0.097
LDL-C	120 (9.0-231.0)	112.0 (18.0-293.0)	0.809
HDL-C	34.0 (3.0-120.0)	48.0 (10.0-144.0)	0.000
TG	156.0 (42.0-1222.0)	146.0 (90.0-2000.0)	0.007
Non-HDL-C	155.0 (15.0-361.0)	146.0 (98.0-451.9)	0.000

Values are shown as mean ± standard deviation, median and interquartile range. LDH: lactate dehydrogenase; CRP: C-reactive protein; WBC: White blood cell; HB: hemoglobin; PLT: platelets; TC: total cholesterol; LDL-C: low-density cholesterol; HDL-C: high-density cholesterol; TG: triacylglycerol; non-HDL-C: non-high density cholesterol.

**Table 3:** – Impact of different variables on the non-surviving group in univariate and multivariate logistic regression analyses

	Univariate				Multivariate			
	OR	%95 CI		p	OR	%95 CI		p
Age	1.655	1.256	2.122	0.000	1.588	1.352	1.755	0.000
LDH	1.004	1.004	1.005	0.000	1.002	1.002	1.003	0.000
CRP	1.010	1.009	1.012	0.000	1.004	1.002	1.006	0.000
WBC	1.057	1.039	1.076	0.000	0.965	0.928	1.003	0.072
TG	1.001	1.000	1.002	0.032	1.005	1.002	1.010	0.03
D-dimer	1.259	1.214	1.306	0.000	1.131	1.088	1.175	0.000
non-HDL-C	1.004	1.002	1.006	0.000	1.005	1.002	1.008	0.001

CI: confidence interval; OR: odds ratio; p: p-value, LDH: lactatedehydrogenase, CRP: C-Reactive Protein; WBC: white blood cell; TG: triglyceride; non-HDL-C:non-high-density cholesterol.

**Table 4:** – Correlation between non-HDL-C and serum biomarkers

N:712		Não HDL-C
Idade	r	0,333
	p	0,001
LDH	r	0,222
	p	0,002
PCR	r	0,235
	p	0,025

LDH: lactatedehydrogenase; CRP: C-reactive protein.

**Figure 1:** – ROC curve analysis of non-HDL-cholesterol. AUC: area under the curve 61.6% sensitivity, 89.2% specificity, cutoff point 150.4

## Discussion

Our study is the first to investigate the association between SARS-COV-2 infection and the non-HDL-C level studied before the COVID-19 disease. The first finding of this study is that the non-HDL-C level studied before the COVID-19 disease is an independent risk factor for mortality. The second important finding is that the non-HDL-C level positively correlates with age, CRP, and LDH.

Similar to previous studies, our study identified age, TG, LDH, CRP, and D-dimer as independent risk factors for COVID-19 infection.^16^ Increased comorbidity, frailty, and immune system disorders that increase with age are considered important factors for the prognosis of COVID-19 infection.^17^ In the study conducted by Onder G et al.^18^ in Italy, the mortality rate in patients hospitalized with COVID-19 infection was 0.4% in those younger than 40 years old, 0.4% in those 50 years old, 3.5% in those 60 years old, 12.8% in those 70 years old, and 20.2% in those 80 years old and older. Although no gender difference was observed between the groups in our study, it has been observed in epidemiological studies that the severity and mortality of COVID-19 infection are higher, especially in older men.^19^In the epidemiological study by Zou F. et al., the average age of the deceased subjects was 56 years, and the majority of them were male (70%).^20^ This situation has many causes, such as metabolic deterioration (increasing obesity, OSAS), hormonal imbalance (decreased testosterone level), increased oxidative and inflammatory mediators (TNF-alpha, IL-1, IL-6), and immune system dysfunction. In addition, epidemiological studies have found that the male patient group’s awareness of the disease and treatment adherence are lower. When concomitant diseases are taken into account, it is found that smoking is more prevalent in the male gender, and the resulting cardiovascular and respiratory concomitant diseases cause high mortality.^21^

CRP resulting from the release of IL-6 is an independent risk factor for both prognosis and mortality of the disease.^22^ Smilowitz et al.,^23^ in their study of 2872 patients, found that the risk of venous thromboembolism increased 2.33 times, the risk of acute kidney failure 2.11 times, and the risk of mortality 2.59 times increased in patients with high CRP. LDH is an intracellular enzyme found in almost all organ systems.^23^ Elevated LDH levels have been observed in COVID-19 infections, increasing disease severity by 6-fold and mortality by 16-fold.^24^D-dimer is an important biomarker of blood coagulation and fibrinolysis that increases significantly in disseminated intravascular coagulation (DIC). Studies have shown a close association between D-dimers and disease severity and mortality. In the study by Zhang L. et al. in hospital-treated patients, mortality was higher in patients with a D-dimer > 2µg/mL with a sensitivity of 92% and a specificity of 83.3%.^25^ Although there was no statistical difference in sodium values between groups in our study, mild hyponatremia was detected in both groups. In a meta-analysis of 23 studies on COVID-19 infections, hyponatremia was observed in 25.8% of 38,753 patients and was more common in patients treated in hospitals and intensive care units.^26^ Habas et al.^27^ reported that hyponatremia is very common in patients with radiological lung infiltration and that the severity of the disease is directly proportional to the depth of hyponatremia.^27^ We think that the reason for the hyponatremia observed in both groups in our study is the patient population. Basically, all patients included in the study were assumed to have radiological lung infiltration and were treated under hospital conditions.

Although non-HDL-C shows an atherogenic burden in recent studies, it has been shown to have predictive and prognostic value for many chronic inflammatory diseases such as coronary artery disease, non-alcoholic hepatosteatosis, OSAS, HIV, and hepatitis B(HBV).^9-13^The inflammatory process begins with forming pro-inflammatory lipid-laden macrophages (foam) by passing through the vascular endothelial layer of non-HDL-C. This leads to lipid peroxidation and the formation of oxygen free radicals. These formed products activate nuclear factor (NF) κB-like transcription factors and cause the release of inflammatory cytokines (TNF-alpha and IL-1B). These mechanisms cause the initiation and progression of vascular inflammation. Numerous studies have reported that atherosclerotic lipid elevation triggers local and systemic inflammation.^28^ In an animal study by Busnelli et al.,^29^ feeding a hypercholesterolemic diet was shown to trigger vascular and chronic systemic inflammation. As a result of the study, in addition to an increase in plasma leukocytes, monocytes, and lymphocytes in many systems such as liver and white adipose tissue, an increase in macrophages and T-cell lymphocytes, as well as an increase in inflammatory cytokines (TNF-alpha, IL-1B, IL-6), were noted.^29^Wang et al.^30^ reported that non-HDL-C is an early marker of vascular endothelial dysfunction in patients with type 2 DM and correlates with CRP.^30^ Prado et al.^31^ showed in their study that non-HDL-C is a better indicator of disease progression and glycemic control than CRP in adolescents and children with type 1 DM.^31^ In the animal study conducted by Poledne et al.,^32^ a positive correlation was found between the development of pro-inflammatory macrophages (CD14-16-36) in visceral adipose tissue, which forms the basis for the development of metabolic syndrome and atherosclerosis, and non-HDL-C levels.^32^Cippollena et al.^33^ found that non-HDL-C was higher in patients who developed restenosis after percutaneous transluminal coronary angioplasty, and there was a positive correlation between restenosis and IL -1B and CRP.^33^ In the study by Karasek et al.,^34^ a positive correlation was observed between non-HDL-C and the inflammatory markers CRP, C-peptide, and PAI (plasminogen activation inhibitor).^34^ According to the results of a 4-year follow-up study in chronic HBV patients, Joo et al.^35^ reported that non-HDL-C was 0.69 times higher in HBsAg-positive patients than in negative patients.^35^

Although no direct study examines the relationship between sepsis and non-HDL-C, PCSK-9 levels, which form the basis of non-HDL-C metabolism, are considered important markers of sepsis. PCSK-9 is a serine protease synthesized mainly in the liver. Its main function is to degrade LDL receptors on the liver surface. In addition, it destroys the apolipoprotein E receptor, Toll-like receptor, VLDL receptor, and LDL-related-1 (LDLR1) protein, which may lead to an abnormal increase in plasma lipoprotein concentration and cytokine levels and thrombosis.^36^ Many studies have found a significant increase in PCSK-9 levels in patients with sepsis and/or septic shock.^37^ Walley et al.^38^ investigated the effect of PCSK-9 loss-of-function (LOF) and gain-of-function on mortality in humans and mice in septic shock. It was determined that LOF in the human group reduced mortality at day 28 (61% versus 71%). In addition, when evaluated after 72 hours in the mice group, it was found that the formation of inflammatory cytokines and endotoxins was lower in the group with LOF.^38^ Boyd et al.^39^ showed elevated PCSK-9 levels during sepsis and increased formation of bacterial endotoxin correlated strongly with multiorgan failure.^39^Although there is no clear information between COVID-19 pneumonia and PCSK-9, it has been predicted that inhibition of PCSK-9 may reduce viral infectivity, as shown by several hypotheses.^40^

In the study by Mostaza et al.,^41^ which examined the prognostic effect of lipid levels before COVID-19 infection, an inverse association was found between a high HDL-C level and mortality in older individuals before the disease.^41^ This is a similar status to our study. In another study by Masana et al.,^42^ serum lipid levels of 1305 patients were examined. At the end of the study, although COVID-19 infection was more severe in patients with high TG and low HDL-C, no prognostic effect of non-HDL-C was observed.^42^ This situation is in contradiction with our study. In our study, high non-HDL-C, together with high TG and low HDL-C, was considered an independent risk factor for mortality. We suspect this is due to the differences in patient population and study method between the studies. In their study, Masana et al.^42^ indicated that CRF, cancer, and DM, considered independent risk factors for mortality in COVID-19 infection, were high in the severe infection group. In addition, non-HDL-C levels and statin use, which have predictive value for mortality in COVID-19 infection, were not investigated between groups. However, our study did not include patients taking statins or patients with cancer and CRF, which are independent risk factors for mortality in COVID-19 infection.

### Limitations

The study’s main limitations are the following: It is based on a single center and retrospective study design, the exact mechanism of this relationship could not be precisely elucidated, and bias may have occurred in the selection of control groups. In addition, lipid changes during patients’ first hospitalization and after treatment could not be examined. Treatments given for COVID-19 infection were not studied. Apolipoprotein-B levels, which indicate total atherogenic burden rather than non-HDL-C, were not measured. Another limitation of the study is that short- and long-term follow-up was not performed.

## Conclusion

Our study has shown for the first time that a higher non-HDL-C level before COVID-19 infection is an independent risk factor for mortality. We believe that the non-HDL-C level studied before COVID-19 infection can be used as a prognostic biomarker for the disease. In addition, we believe that it can help us understand the pathophysiology and develop new treatment strategies. Further prospective studies with large samples are warranted to understand better the pathogenesis of COVID-19 and the diagnostic and therapeutic value of non-HDL-C in COVID-19 patients.
